# Keep it real: rethinking the primacy of experimental control in cognitive neuroscience

**DOI:** 10.1016/j.neuroimage.2020.117254

**Published:** 2020-08-13

**Authors:** Samuel A. Nastase, Ariel Goldstein, Uri Hasson

**Affiliations:** a Princeton Neuroscience Institute, Princeton University, Princeton, NJ, USA; b Department of Psychology, Princeton University, Princeton, NJ, USA

**Keywords:** Ecological psychology, Ecological validity, Experimental design, Generalizability, Naturalistic stimuli, Representative design

## Abstract

Naturalistic experimental paradigms in neuroimaging arose from a pressure to test the validity of models we derive from highly-controlled experiments in real-world contexts. In many cases, however, such efforts led to the realization that models developed under particular experimental manipulations failed to capture much variance outside the context of that manipulation. The critique of non-naturalistic experiments is not a recent development; it echoes a persistent and subversive thread in the history of modern psychology. The brain has evolved to guide behavior in a multidimensional world with many interacting variables. The assumption that artificially decoupling and manipulating these variables will lead to a satisfactory understanding of the brain may be untenable. We develop an argument for the primacy of naturalistic paradigms, and point to recent developments in machine learning as an example of the transformative power of relinquishing control. Naturalistic paradigms should not be deployed as an afterthought if we hope to build models of brain and behavior that extend beyond the laboratory into the real world.

Cognitive neuroscientists employ clever experimental manipulations in hopes of discovering interpretable relationships between brain, behavior, and the environment. There is a commitment—often implicit—in both our scientific thinking and writing that the models we derive from tightly-controlled experimental manipulations will provide some traction in real-world contexts. This commitment relies on the assumption that the human brain implements a set of nomothetic principles or rules that capture the underlying principles or rules by which the world works. We assume that these rules, like in classical physics, are relatively simple and interpretable, and, once discovered, will extrapolate to the richness of human behavior (Jolly and Chang, 2019). We proceed by filtering out as many seemingly irrelevant variables (considered “confounds” or “noise”) as possible in hopes of isolating the handful of latent variables (considered “signal”) dictating brain—behavior relationships. To what extent do our models actually generalize outside the laboratory? What proportion of neural or behavioral variability do our models predict in real-life contexts? These kinds of questions have prompted the neuroimaging community, and neuroscience more broadly, to begin adopting more naturalistic experimental paradigms (Hasson and Honey, 2012; Maguire, 2012; Hamilton and Huth, 2018; Matusz et al., 2019; Sonkusare et al., 2019).

Naturalistic paradigms have generally been considered a testbed for models developed under highly-controlled experimental paradigms. In neuroimaging, naturalistic stimuli were introduced optimistically in hopes of validating existing models (Bartels and Zeki, 2004; Hasson et al., 2004). This optimism has declined over the intervening years. In the following, we provide a historical context for naturalistic neuroimaging and appeal to representative design as a principled basis for ecological generalizability (Brunswik, 1947). We assume that no cognitive neuroscientist would be satisfied with a science strictly confined to peculiar experimental manipulations with little relevance outside the laboratory. However, the world outside the laboratory is not amenable to many of the assumptions of classical experimental design; real-world ecological variables are often multidimensional, sometimes nonlinear, and interact in unexpected ways. To make matters worse, evolution has built a brain that capitalizes on these interactions to guide adaptive behavior.

To be clear, we are not arguing indiscriminately against controlled experiments. Experimental manipulations provide a powerful and necessary tool for testing hypotheses and models. Our argument pertains to the source and character of these hypotheses. As experimentalists, we take complex phenomena and try to deconstruct them into manageable components that we can more easily manipulate in our experiments. We often bootstrap hypotheses from preexisting experimental manipulations, thus superimposing the assumptions of experimental design on the process of hypothesis formation and data generation. When data from the experimental manipulation adjudicate in favor of the hypothesis, we generally assume that we have discovered something meaningful about brain and behavior. However, when stringent design considerations constrict both hypotheses and data, we risk maneuvering ourselves into theoretical corners that are difficult to reconcile with ecological brain function. We argue that this necessitates a shift toward the primacy of naturalistic paradigms in developing and evaluating models of brain and behavior.

## 1. What problems does the brain confront outside the laboratory?

Evolution has shaped our brains to guide behavior in a multidimensional, uncertain world. The importance of this fact has been periodically reasserted in the schools of functional and ecological psychology (e.g., Brunswik, 1943; Gibson, 1979), but the implications remain underappreciated. We contend that many properties of the brain, as an evolutionary solution for guiding adaptive behavior, undermine many of the theoretical assumptions of cognitive neuroscience outlined above (see Hasson et al., 2020, for an extended discussion). Evolution does not have the privilege of operating under controlled laboratory conditions, does not necessarily produce intuitively “optimal” solutions (cf. Attneave, 1954; Barlow, 1961; Olshausen and Field, 1996; Lewicki, 2002), and does not appeal to human-interpretable design principles (Dennett, 1995; Cisek, 2019). In the case of the brain, evolution has converged on a high-dimensional modeling/control organ for estimating whatever structure in the world is relevant for guiding context-specific adaptive behavior. In this respect, the brain does not operate like a scientist, as the kind of estimation needed to guide behavior does not necessitate the kind of understanding scientists seek. In other words, the brain is not necessarily designed to rely on simple, human-interpretable variables; it does not always cleanly segregate variables into signal and noise; and it does not necessarily respect the theoretical boundaries imposed by our experimental designs.

Ecological variables in the environment are poorly understood. Any ecologically relevant “signal” in the environment is multidimensional and there are nonlinearities and interactions among dimensions (Campbell, 1973; Cronbach, 1975; Gibson, 1979). Furthermore, ecologically relevant dimensions of the environment are always mixed with non-relevant dimensions. The brain cannot simply ignore non-relevant dimensions; it must learn to actively adjust particular dimensions in order to guide behavior. In most ecological situations, the relevant dimensions for a particular action (e.g., recognizing a face, or interpreting the meaning of words in a particular context) are always mixed with non-relevant dimensions (e.g., luminance, motion, or occlusion of the face; the sentence structure used or the accent of a speaker). To perform these tasks, the brain must dynamically weight and re-weight all the incoming dimensions as a function of task and context. In other words, there are no two systems such that one processes the “signal” and the other processes “confounds” or “noise.” Classical controlled experiments, where the vast majority of these variables are artificially clamped or factored out, ignore one of the central problems the brain must face, and may hinder our understanding of the solutions the brain has found to overcome it. It is surprisingly difficult to generalize from a contrived experiment artificially isolating a handful of experimental variables to other contexts with five, ten, or perhaps hundreds of dimensions; however this doesn’t discourage us for interpreting experimental results more generally (Cronbach et al., 1963; Yarkoni, 2019).

Take for example the seminal findings of Hubel and Wiesel (1962): probing the visual system of the anaesthetized cat with differently oriented edges reveals an orderly model of orientation tuning in primary visual cortex. It was thought that extending this systematic program to other stimulus features would eventually allow us to piece together a complete model of early visual function. However, despite revealing some important insights, the limits of this program have become increasingly evident. For example, work by David et al. (2004) has demonstrated that the spatiotemporal tuning of neurons in primary visual cortex (V1) differs substantially between naturalistic and non-naturalistic contexts, likely due to nonlinear relationships among neural variables and environmental variables. Models of neural tuning derived from synthetic stimuli in the vein of Hubel and Wiesel may not generalize well to the real-world conditions in which our brains evolved (Simoncelli and Olshausen, 2001; Kayser et al., 2004; Felsen and Dan, 2005; McMahon et al., 2015; Park et al., 2017; Leopold and Park, 2020). Olshausen and Field (2005), famously cautioned that “we can rightfully claim to understand only 10% to 20% of how V1 actually operates under normal conditions,” attributing this in part to biased stimulus sampling and a tendency toward easily-interpretable models.

## 2. Systematic and representative design

Advocates for naturalistic paradigms often appeal to their “ecological validity,” a term that originated with Egon Brunswik (Brunswik, 1947, 1949).^1^ Brunswik championed a heterodox school of psychological theory summarized as “probabilistic functionalism,” emphasizing the messy, probabilistic nature of organism–environment relations and the importance of Darwin’s notion of adaptive fitness in guiding behavior (Tolman and Brunswik, 1935; Brunswik, 1943). Brunswik (1949) contended that psychology maintains a “double standard” in the application of sampling theory (Neyman, 1934; Kruskal and Mosteller, 1980) to subjects and stimuli: whereas subjects are sampled with the goal of generalizing to the population, stimuli and tasks generally are not.

Brunswik challenged the paradigm of “systematic design”—the practice of artificially reducing the world to a small number of hand-picked variables for experimental manipulation—on grounds that it often fails to actually isolate variables of interest and tends to impose non-naturalistic relationships among variables (Brunswik, 1955). In contrast, Brunswik advocated for “representative design,” arguing that we should sample stimuli or conditions in a way that respects the distribution and covariance of ecological variables if we hope to achieve generalizability beyond the boundaries of the experimental manipulation. Ecological generalizability demands a “representative sampling of situations” where “situational instances in an ecology are analogous to individuals in a population” (Brunswik, 1955, p. 198). Ecologically relevant configurations of variables carve out a manifold in a multidimensional space of organism—environment relations. Systematic experimental manipulations that clamp or orthogonalize certain variables risk unintentionally relocating an experiment off the manifold into a peculiar region of this space, thus for feiting ecological generalizability.

Though considered heretical during Brunswik’s lifetime, the critical thrust of his program has nonetheless permeated a variety of fields (Hammond, 1955; Jenkins, 1974; Bronfenbrenner, 1977; Neisser and Hyman, 2000; Fiedler, 2011). For example, Barker’s (1965) “ecological psychology” advocates for the psychologist as a “transducer” of psychological phenomena *in situ*, rather than the traditional "operator/transducer” who manipulates the environment and organism to “send messages to [them]self.” This critique also resonates with modern statistical debates: for example, the “stimulus-as-fixed-effect” controversy in psycholinguistics (Coleman, 1964; Clark, 1973; Baayen et al., 2008), social psychology (Wells and Windschitl, 1999; Judd et al., 2012), and neuroimaging (Bedny et al., 2007; Westfall et al., 2016); or endogenous selection bias, where a spurious relationship between variables of interest is induced by biased sampling along another collider variable (Elwert and Winship, 2014). One particular zenith along this line of thought was Gibson’s (1979) theory of “direct perception,” which forcefully elevated the environment itself to a principal object of study in psychology, emphasizing in particular the organism- and context-specific elements of the environment that offer opportunities for adaptive behavior (i.e., “affordances”). Despite the artificiality of many laboratory manipulations, an organism cannot be decoupled from the environment in which it evolved (von Uexküll, 1934; Chiel and Beer, 1997; Gomez-Marin and Ghazanfar, 2019).

Much of cognitive neuroscience still operates in a similar regime to Hubel and Wiesel, using contrived, non-naturalistic stimuli and tasks in hopes of revealing fundamental features of functional neuroanatomy. This analytic, reductionist program is endemic to psychology and neuroscience more broadly: complex, real-world phenomena are decomposed into increasingly circumscribed subcomponents that are manifest in highly-constrained experimental manipulations (cf. Braitenberg, 1984; Cisek, 2019).^2^ We use disjoint tasks to devise complex taxonomies of memory (e.g., Squire, 2004) and attention (e.g., Carrasco, 2011), subdividing the brain into a mosaic of regions reflecting intuitive, hand-picked contrasts (e.g., Kanwisher et al., 1997; Kanwisher, 2010); but rarely do we reassemble these manipulations into functional, ecological behavior. How do these disparate systems conspire to perform complex, real-world behavior (e.g., summarizing a complex idea and verbally conveying it to a colleague, a task many readers perform every day)? The assumption that we can someday cobble together these piecemeal processes and representations into a satisfying model of brain and behavior is tenuous at best (Newell, 1973; Meehl, 1990). Concerns about the utility of traditional laboratory tasks are not specific to neural measurements (e.g., Elliott et al., 2020); in fact there is increasing evidence that many behavioral tasks have little conceptual overlap with self-report measures and fail to capture real-world behavior (e.g., Eisenberg et al., 2019; Dang et al., 2020).

To illustrate this point, consider working memory processes in a daily context, such as reading a story, as opposed to a laboratory context, such as a delayed match-to-sample task. In the delayed match-to-sample task, the process of protecting information in a working memory buffer is isolated from other perceptual, decision-making, and motor-related processes by the structure of the task itself. However, in real-world contexts, each word we accumulate while reading a story interacts with and is synthesized with all previous written or spoken words in an evolving narrative (Willems et al., 2020). The naturalistic reading task reveals that neural systems, across all levels of the processing hierarchy, need to accumulate, maintain, and synthesize information at their preferred processing timescale, making the classical distinction between processing systems and memory systems intangible (see Hasson et al., 2015). Face perception provides another illustrative example. The first step in studying face perception is typically to experimentally strip away cumbersome social content like facial expressions, personal familiarity, and temporal dynamics. Tightly-constrained stimulus parameterization and contrasts with randomized trial order reveals orderly face-selective responses in several cortical areas (e.g., Kanwisher et al., 1997; Tsao et al., 2006). However, the dynamics of face perception circuitry become considerably more nuanced when presented with complex, naturalistic stimuli, particularly in social contexts (McMahon et al., 2015; Russ and Leopold, 2015; Park et al., 2017; Leopold and Park, 2020). Cortical areas with seemingly uniform face-selective responses presumably also encode dynamic features that were simply not present in the decontextualized, static face stimuli. In this sense, naturalistic stimuli—in which faces are persistent, sometimes familiar, and carry dynamic social and semantic content—allow us to better gauge the relative contributions of different variables, and can reveal the importance of previously underappreciated variables for neural representation (Haxby et al., 2020). Beyond naturalistic stimuli, there is also evidence that spontaneous, naturalistic behavior plays an unexpectedly large role in neural activity throughout the brain, including in putative low-level sensory areas (e.g., Musall et al., 2019; Stringer et al., 2019).

## 3. Lessons from machine learning

Recent advances in artificial neural networks (ANNs) provide an instructive foil for experimental neuroscience. The machine learning community has made tremendous strides in building neurally-inspired models that match or exceed human performance in cognitive tasks spanning visual processing, language processing, and complex gameplay (LeCun et al., 2015). Why have neural network models developed in the machine learning community so dramatically outstripped models developed in psychology and neuroscience laboratories?

One of the key developments was to relinquish some amount of control and embrace the complexity of real life. The machine learning ing community does not fixate on “experimental design” in the way that neuroscientists do. They do not manufacture a small set of well-behaved inputs in developing their models; instead, they use vast, largely-unconstrained training data sampled from the real world. They do not impose the strong constraint that their models must learn human-interpretable representations or rules. Instead, machine learning has—for pragmatic reasons—prioritized predictive power over easily-interpretable, explanatory models (Breiman, 2001; Yarkoni and Westfall, 2017; Varoquaux and Poldrack, 2019). The implicit goal in most cases is not to model an experimentally-isolated cognitive process, but to build useful models of the phenomenon of interest out in the world. Take for example a deep convolutional neural network for face recognition that matches (and exceeds) human performance in recognizing face identities (Schroff et al., 2015). This model is trained on face images ages spanning numerous identities sampled “in the wild” to include all manner of naturalistic “confounds”—differences in expression, lighting, head angle, and so on. The same model trained on a tightly-controlled subset of facial images would fail dramatically due to biased, non-representative sampling (O’Toole et al., 2018; Srivastava and Grill-Spector, 2018).

The way these models learn to map noisy, real-world inputs along objective functions to perform complex tasks resonates with Gibson’s (1979) notions of direct perception. Much like the brain, the structure of the fitted model is inseparable from the task(s) the model is trained to perform in the world. We argue that the way both artificial and biological neural networks learn to pursue objective functions cleaves more toward Gibson’s (1979) notion of direct perception than, for example, Marr’s (1982) constructivist, representationalist approach (Brooks, 1991; Pezzulo and Cisek, 2016; Hasson et al., 2020). In the same way that evolutionary theory shifted our understanding of biology to a few relatively simple processes and principles, the effectiveness of artificial neural networks in learning cognitive tasks may force us to rethink the neural code (see Richards et al., 2019, and Hasson et al., 2020). The recent success of neural networks in solving many of the tasks we study in cognitive neuroscience serves as a cautionary tale for those probing the brain for easily-interpretable representations.

## 4. Studying ecological brain function without losing control

Most psychologists and neuroscientists are trained to respect the primacy of experimental control. We celebrate the ingenuity of tasks that manage to isolate a handful of interpretable variables from confounds. When a particular task or manipulation fails to elicit the desired effect, we often adjust the task or fine-tune the manipulation in hopes of homing in on the effect. This research program hinges on the assumption that the brain extrapolates from a number of human-interpretable representations and processes to navigate the world; and that using clever designs to experimentally isolate the neural implementation of these rules will allow us to extrapolate to ecological behavior. With these assumptions in hand, we consider tightly-controlled experimental manipulations as the principal (perhaps only) source of insight into the underlying neural code (Gillis and Schneider, 1966), whereas naturalistic paradigms are treated as a necessary (albeit inconvenient) testbed for validating these theories. But what if these assumptions are unsound? What if nonlinearities and interactions among environmental variables hamstring generalization from contrived experiments? What if biological systems rely more on exhaustive sampling and brute-force interpolation rather than rule-based extrapolation? How is the cognitive neuroscientist to proceed?

Naturalistic paradigms are not a panacea and are not trivial to implement or analyze. We do believe there is value in using controlled experiments to test hypotheses, but contend that these hypotheses should stem from ecological considerations and address head-on the actual problems the brain confronts in the world. Controlled experiments can reveal important boundary conditions of ecological brain function, and no single paradigm can be exhaustively representative or generalizable. However, we believe that non-naturalistic experimental manipulations have occupied an overly privileged position in cognitive neuroscience.

We caution against allowing classical experimental manipulations to play an outsized role in hypothesis formation. For example, if the goal is to differentiate neural systems processing articulatory and semantic features of words, rather than using tightly-controlled lists of words and nonwords, we recommend using natural speech stimuli and comparing models of articulation and semantic content (e.g., de Heer et al., 2017). When designing an experiment, we recommend, whenever possible, to use naturalistic tasks and to sample stimuli and conditions (including controls) from ecological contexts; for example, leveraging each subject’s personal social network (Parkinson et al., 2017, 2018; Hyon et al., 2020), probing memory using naturalistic recall (Chen et al., 2017; Zadbood et al., 2017; Heusser et al., 2018), comparing natural language across modalities and contexts (Stephens et al., 2010; Regev et al., 2013; Yeshurun et al., 2017; Deniz et al., 2019), and using data-driven modeling to capture the complexity of naturalistic neural responses (Haxby et al., 2011, 2020; Baldassano et al., 2017; Chang et al., 2018; Nastase et al., 2019). Appealing to representative sampling in experimental design will tend to introduce (ecological) intercorrelations among variables, and may reduce statistical power for low-frequency phenomena (Hamilton and Huth, 2018); in this sense, naturalistic paradigms may resemble observational research, and may benefit from the associated methods (e.g., Rohrer, 2018). Our thesis, however, is that anyone adopting the alternative approach—clamping or and artificially orthogonalizing these variables—must contend with the challenge of ecological generalizability.

Building a more ecological research program demands increasingly rich data and quantitative tools for describing brain, behavior, and environment. Publicly shared naturalistic datasets (e.g., Hanke et al., 2014; Nastase et al., 2019) have exceptional re-use value and can serve as benchmarks for model comparison (DuPre et al., 2019). These datasets will eventually become exhausted as competing models improve and reach ceiling performance; data generators will never be out of work and there will always be a market for innovations in data acquisition. Developing technologies, such as continuous intracranial electroencephalography (iEEG; e.g., Wang et al., 2016), functional near-infrared spectroscopy (fNIRS; e.g., Liu et al., 2017), high-density diffuse optical tomography (HD-DOT; e.g., Fishell et al., 2019), and wearable magnetoencephalography (MEG; Boto et al., 2018) promise higher-fidelity and more ergonomic neuroimaging. Even the workhorse fMRI is beginning to see increased adoption of immersive virtual reality paradigms (Mathiak and Weber, 2006; Spiers and Maguire, 2006, 2007; Maguire, 2012). Finally, we are seeing advances in quantifying the richness of natural behavior (Gomez-Marin et al., 2014; Calhoun and Murthy, 2017; Nath et al., 2019; Pereira et al., 2019). We live in an age of ubiquitous real-life behavioral data collection (for better or worse); experience sampling technologies such as mobile sensing (Miller, 2012; Harari et al., 2016) provide new windows into naturalistic behavior, and can be used to procure subject-specific representative stimuli (Nielson et al., 2015; Rissman et al., 2016).

In the context of representative design, Brunswik contends that the “challenge of further [isolating variables] must be met by after-the-fact, mathematical means” (Brunswik, 1955, pp. 202–203). This resonates with the more recent notion of “late commitment” in cognitive neuroscience (Kriegeskorte et al., 2008, p. 19), wherein theoretical assumptions are relaxed at the stage of experimental design and data collection, and later imposed at the analysis stage. Representative design is also conducive to a “system identification” approach for mapping between formal models of the environment and neural responses (Wu et al., 2006; Naselaris et al., 2011; Gallant et al., 2012; Nunez-Elizalde et al., 2019). In this framework, explicit models capturing, e.g., visual or semantic content (Nishimoto et al., 2011; Huth et al., 2012, 2016) are constructed to predict brain activity from naturalistic stimuli or tasks. In both of these frameworks, hypotheses are formalized as explicit models of the stimulus or task, and the relative quality of a given model is quantified in terms of its accuracy in predicting neural responses to novel input. Commonality analysis (Mood, 1971; Seibold and McPhee, 1979) provides a statistical framework for partitioning variance due to combinations of variables and has been deployed for both voxelwise encoding models (e.g., Lescroart et al., 2015; de Heer et al., 2017) and pattern-based representational similarity analysis (e.g., Groen et al., 2018; Hebart et al., 2018). Adopting a prediction-oriented framework with an emphasis on accounting for variance in real-world contexts may help combat the reductionism inherent in contrived experimental manipulations and simple models (Yarkoni and Westfall, 2017; Varoquaux and Poldrack, 2019).

We can summarize these examples into several concrete recommendations, many of which are reflected in the exceptional body of work presented in this special issue: (1) formulate hypotheses with ecological considerations in mind; (2) rather than constraining data collection, sample brain activity under representative contexts for the ecological behaviors you wish to study; (3) find manipulations for characterizing the boundary conditions that naturally emerge in real-life contexts; (4) when possible, formalize hypotheses as explicit models capable of making quantitative predictions of neural activity under the most naturalistic conditions possible; (5) interrogate your models with the goal of understanding not only the neural data, but also the structure of the task, stimulus, or environment; (6) use your insights to generate new predictions to be tested in real-life contexts or under more controlled conditions as necessary.

## 5. Conclusion

We hope our argument has punctuated the fundamental tension between experimental control and ecological generalizability. We cannot naively decompose organism—environment relations into contrived experimental manipulations in hopes of recomposing them into a satisfying understanding of ecological brain function. By dogmatically adhering to systematic design, we risk creating a cognitive neuroscience of contrived experimental manipulations that have little meaning outside the laboratory—confining ourselves to what Brunswik (1947, p. 110) referred to as “a self-created ivory-tower ecology.” Naturalistic paradigms should not be relegated to post hoc model validation—they should provide a foundation from which theories are developed (Hasson et al., 2020). Moving toward a more ecological cognitive neuroscience is not simply a matter of plugging more realistic stimuli into our usual experiments, but stepping outside our usual mode of inquiry and reframing our questions to encompass the nested dynamics of brain, body, and environment (Gomez-Marin and Ghazanfar, 2019). We are optimistic that adopting an ecological perspective will not only complement our existing models, but revolutionize them.

## Footnotes

1 Brunswik in fact used the term “ecological validity” in a narrow sense to indicate the utility of a perceptual cue with respect to an ecologically-relevant state of the environment—modern usage more closely resembles Brunswik’s notion of “representative design” (Hammond and Stewart, 2001; Araújo et al., 2007).

2 Bannister (1966) humorously put it: “In order to behave like scientists, [experimental psychologists] must construct situations in which our subjects are totally controlled, manipulated and measured. We must cut our subjects down to size. We construct situations in which they can behave as little like human beings as possible and we do this in order to allow ourselves to make statements about the nature of their humanity.”
